# Cost of Cholera for Households and Health Facilities, Somalia

**DOI:** 10.1007/s44197-024-00278-6

**Published:** 2024-07-18

**Authors:** Salvador Figuereo, Ian Yoon, Ssentamu Simon Kaddu, Mutaawe Lubogo, Joaquin Baruch, Asm Amjad Hossain, Sahra Isse Mohamed, Ali H. A. Abubakar, Khalid Mohamed Mohamud, Sk Md Mamunur Rahman Malik

**Affiliations:** 1 World Health Organization, Somalia Country Office, Mogadishu, Somalia; 2 World Health Organization, Headquarters, Geneva, Switzerland; 3 Ministry of Health, Mogadishu, Somalia; 4 World Health Organization, Eastern Mediterranean Regional Office, Cairo, Egypt

**Keywords:** Cholera, Cost of Illness, Family and Household, Health Facilities, Somalia

## Abstract

**Introduction:**

Cholera remains a substantial public health challenge in Somalia. Ongoing droughts in the country have caused significant outbreaks which have negatively affected the lives of many individuals and overwhelmed health facilities. We aimed to estimate the costs associated with cholera cases for households and health facilities in Somalia.

**Methods:**

This cost-of-illness study was conducted in five cholera treatment centres in Somalia and 400 patients treated in these facilities. Data collection took place during October and November 2023. Given that a significant portion of the patients were children, we interviewed their caregivers to gather cost data. We interviewed staff at the centres and the patients. The data obtained from the household questionnaire covered direct (medical and non-medical) and indirect (lost wages) costs, while direct costs were estimated for the health facility (personnel salaries, drugs and consumables used to treat a patient, and utility expenses). All costs were calculated in US dollars (USD), using 2023 as the base year for the estimation.

**Results:**

The average total cost of a cholera episode for a household was US$ 33.94 (2023 USD), with 50.4% (US$ 17.12) being direct costs and 49.6% (US$ 16.82) indirect costs. The average total cost for a health facility to treat an episode of cholera was US$ 82.65. The overall average cost to households and health facilities was US$ 116.59. The average length of stay for a patient was 3.08 days. In the households, patients aged 41 years and older incurred the highest mean total cost (US$ 73.90) while patients younger than 5 years had the lowest cost (US$ 21.02). Additionally, 61.8% of households had to use family savings to cover the cost of the cholera episode, while 14.5% had to borrow money. Most patients (71.8%) were younger than 16 years– 45.3% were 5 years or younger– and 94.0% had never received a cholera vaccine.

**Conclusion:**

Our study suggests that preventing one cholera episode in Somalia could avert substantial losses for both the households and cholera treatment centres. The findings shed light on the expenses associated with cholera that extend beyond healthcare, including substantial direct and indirect costs borne by households. Preventing cholera cases could lead to a decrease in this economic burden, consequently our study supports the need for preventive measures.

**Supplementary Information:**

The online version contains supplementary material available at 10.1007/s44197-024-00278-6.

## Introduction

Cholera is an intestinal disease usually spread through water and food that is contaminated with the bacterium *Vibrio cholerae* [1, 2]. This disease is often associated with limited access to clean water and safe sanitation. In 2022, 472 697 cholera cases were reported worldwide, more than double the cases reported in 2021 (223 370). The number of countries reporting cholera cases also increased from 35 to 44 countries in the same period [3]. Cholera remains a public health threat in the countries of sub-Saharan Africa where an estimated 140 000 suspected cases are reported every year from these countries of both endemic and epidemic settings making it one of the regions with the highest cholera burden [4]. Even though the countries in sub-Saharan Africa suffer a high disproportionate burden of cholera, the national-level economic burden of cholera, disaggregated by different sectors, occupations of patients and relatives, are absent in most of these countries [5, 6]. Children younger than 5 years are the most affected population groups in most of these countries [6, 7]. This makes it important for the countries to prevent and control cholera and other associated diarrhoeal diseases illness in children as persistent diarrhoeal illness has detrimental effects beyond the duration of illness in children such as being associated with malnutrition and stunted growth which can affect productivity and loss of income of these children in the later life [8]. Somalia is a cholera-endemic country and has reported uninterrupted transmission since the drought in 2017 [9]. Cholera outbreaks in Somalia are exacerbated by the substantial displacement of the population due to conflict, droughts and floods– an estimated 3.86 million persons were internally displaced throughout Somalia as of the end of 2022 [10].

In response to the recurrent cholera outbreaks in Somalia, the World Health Organization (WHO) country office in Somalia, in collaboration with the Ministry of Health of Somalia and other development partners, launched a comprehensive cholera response plan. This included timely data collection, case management support, improving access to safe water and conducting both reactive and preventive oral cholera vaccine (OCV) campaigns in regular intervals [11].

In Somalia, cholera outbreaks usually coincide with the Gu rains (April–June) and Deyr rains (Oct–Dec) with the Deyr season producing most of the case load of suspected cholera. Treatment for suspected cases is provided through establishing Cholera Treatment Centres (CTCs); these facilities provide specialized care exclusively for cholera patients. While CTCs handle both inpatient and outpatient cases, most of the patients with moderate to severe dehydration are admitted at the CTCs while the cases with mild dehydration are managed at the outpatient level. The inpatient services are provided for severe cholera cases requiring extensive medical intervention (intravenous infusion and antibiotics for severe cases and patients with medical complications, if needed), while outpatient care is available for milder cases that can be managed with oral rehydration solutions (ORS) and minimal medication. In terms of cost for establishing and managing patient care at the CTCs, the Ministry of Health, supported by international aid agencies, cover primary medical expenses, including medications, salaries of care givers, diagnostics, hospitalization and utilities at these facilities. However, even when the treatment is free of cost for the patients, households often bear additional costs. These include any treatment before visiting the CTC and non-medical costs, such as transportation to the centres and indirect costs like forgone wages for the patients and their caregivers. In a country like Somalia, where financial resources are limited, an estimated 55% of the Somali population lived below the national poverty line in 2022 [12] and many are affected by humanitarian crisis, armed conflict and displacement, these costs represent a significant economic burden on the families.

Inadequate handwashing, lack of access to clean drinking water and absence of proper latrines are indeed major determinants of cholera outbreaks in many regions around the world [13]. By implementing a multifaceted approach combining OCV campaigns with interventions such as water, sanitation and hygiene (WASH), risk communication, community engagement, and surveillance and early detection the number of cholera cases in Somalia has remained at fewer than 20 000 since 2018 [11]. Nonetheless, the threat of a cholera outbreak is still a serious public health challenge in a resource constrained and humanitarian crisis settings in Somalia.

Cholera outbreaks have serious repercussions in terms of morbidity and mortality. They also have economic and social consequences. The cost of cholera treatment imposes a considerable economic burden on Africa. Across 44 African countries 71 126 cases and 937 deaths were reported to WHO in 2015, resulting in an estimated economic burden of US$ 68 million from cholera-related illness and treatment [14]. Addressing the economic burden of cholera requires a clear understanding of the costs involved in treating and preventing the disease. This study focuses on quantifying the costs of treating this disease in the context of Somalia, where the financial impact on households and health facilities is not properly documented.

Several countries such as Bangladesh [15], Ghana [16] and Zanzibar [17] have assessed the economic repercussions of cholera through cost-of-illness analyses. These analyses have contributed to more effective prevention and control strategies by making a case for WASH interventions, vaccination programmes, improved healthcare facilities and advocacy for increased funding for cholera prevention and control programmes. Despite the high cholera incidence in Somalia, the cost of treating a cholera case as well as other financial implications are not known. Without data on the economic burden of cholera, the Ministry of Health and its partners face substantial challenges in their efforts against preventing cholera and meeting the global goal of eliminating cholera by 2030.

Therefore, this study aims to fill that knowledge gap by providing detailed estimates of the costs borne by families and health facilities for treatment of cholera in Somalia. Understanding the economic consequences of cholera in Somalia would help inform planning for prevention and control strategies and effective use of resources. With this study we aim to estimate the cost of a cholera case in Somalia for a better understanding of the economic offsets that can be achieved in the country through better prevention and control strategies and use it as evidence to make a case for increased investment for cholera control in a humanitarian crisis setting. To our knowledge, such information or data are not available from countries with acute or protracted humanitarian crisis. Therefore, we also hope to provide a basis for conducting national level economic burden and evaluations of cholera control in other countries with a similar context.

## Methods

### Study Setting and Sample

We purposefully selected five cholera treatment centres in five districts of Somalia: Baidoa, Banadir (Mogadishu), Belethawo, Jowhar and Kismayo. These districts had the highest incidence of cholera at the time of the study and were accessible by the research team. Owing to insecurity prevailing in many parts of the country due to armed conflicts, many areas of the country were not accessible. In selected districts, the cholera outbreak was laboratory confirmed by regional public health laboratories through stool culture. According to national surveillance guidelines, all cases that presented with three or more loose motions in 24 h from the same location were classified as suspected case of cholera.

These five CTCs, providing both outpatient and inpatient care, were chosen for the study based on caseload to provide a large number of cholera cases for inclusion in the analysis. Additionally, given the ongoing challenges in the conflict-affected regions of the country, we considered those facilities which were accessible in order to minimize risks and ensure the safety of the data collectors. Our study estimated the costs associated with both perspectives, the **health facilities** and the **households**. This comprehensive approach captured a broad range of costs associated with cholera treatment focusing on the financial costs of both perspectives while also incorporating elements of economic costs analysis by including indirect costs, such as forgone wages and loss of productivity borne by the households.

To estimate health facility costs, health workers in the selected facilities were interviewed by trained data collectors using a questionnaire to estimate the following: expenses incurred by the facility (e.g. utilities); the cost of treating a cholera patient; and the number of cholera cases treated in each facility. All data were validated through expert consultation to ensure the validity of the information obtained.

To estimate the household cost, our inclusion criteria included the random selection of study participants (cholera patients) from the list of cholera patients treated in each of the five selected cholera treatment centres during September and October 2023. The study participants had been confirmed for cholera by either a rapid diagnostic test or stool culture and had completed their treatment at the health facility. We conducted interviews 15–20 days after discharge to allow the patients to complete their treatment and for all the costs incurred while sick to be considered.

Additionally, patients (or their caretakers) provided informed consent to participate in the study. Patients who did not consent or were not available were excluded. Interviews were done by the trained data collectors at the patient’s home and usually with the head of the family or the person in the family most familiar with the cost incurred during the patient’s cholera treatment. Interviews took place during the months of October and November 2023.

We based our sample size on Kish Leslie’s formula [18], considering a probability of 50% and precision of 5% to get the maximum number of possible cases to include in the study. We included a total of 400 patients (including 20 patients for incomplete or non-response) with complete records in national cholera line list for our study. Selected patients were randomly selected by randomizing and sorting out the list of cholera patients and drawn proportionately. The quota allocation for each district was calculated as a proportionate sample of the total caseload. From the 400 patients 240 were from the cholera treatment centre in Banadir and 40 each were drawn from the four remaining cholera treatment centres. This sample size is consistent with our literature review and similar to the other studies, we did not associate the cost of cholera with a confidence interval.

### Data Collection

We used Open Data Kit (ODK) [19] for data collection. Data collection forms were designed based on the questionnaires and customized to meet the specific requirements of our study. These forms were preloaded onto handheld tablets for use in the interviews. Training of research assistants and pilot testing of the tools to ensure data quality were conducted before starting the data collection phase. The use of mobile data collection technology streamlined the process and ensured the accuracy and completeness of the information obtained, enhancing the overall quality of our dataset. Data collection was carried out from October to November 2023.

### Questionnaires

To estimate the cost of cholera, we used a similar approach as cost-of-illness studies conducted in Malawi [20] and Zambia [21]. We used two separate questionnaires to gather information from households and health care facilities (see supplementary material).

The household questionnaire asked about the direct and indirect costs incurred by the households, including expenses related to medical treatment, transportation and accommodation, and the economic repercussions of cholera on employment, productivity and caretakers. Socioeconomic characteristics of the patients were also recorded. We also asked about the strategies the household had used to cope with the expense of the cholera episode.

The health facility questionnaire collected information on facility operational costs and the resources needed to treat cholera cases effectively. To completely assess the cost of treating cholera in health facilities, we had to account for the costs met by WHO, partners and other donors, who cover the operational costs of running the facilities, such as, salaries of the health facility personnel, the medicines and consumables used when treating a patient, and the regular cost of utilities at the health facilities, namely electricity and water.

### Household Costs

To estimate the overall cost of a cholera episode, we categorized costs into direct and indirect costs for the households. We split direct costs into direct medical costs and direct non-medical costs.

Direct medical costs included: **Consultations**, **diagnostics tests**, **medicine** and **hospitalization**. It is important to highlight that typically these costs are provided free of charge at the CTCs in Somalia. However, the costs inquired for in this section correspond to those additional services not covered by the facilities and the expenses incurred by the patients before and/or after visiting the CTCs.

**Consultations**, covering costs associated with physician consultation, medical advice and expertise provided by a health care professional before or after arriving at the health centre. **Diagnostic tests** were the costs associated with laboratory testing to diagnose the cholera episode. Medication and other drugs encompass the cost of medicine, including drugs for the treatment of cholera and other necessary medications additional to those provided at the health facility. Hospitalization include the costs of intensive or additional care in a hospital or health care facility for further treatment.

Direct non-medical costs included: transportation, consumables and dietary requirements.

**Transportation costs** cover the estimated expenses for the patient (and its caregiver if any) to reach the health care facility, given that many patients in our sample were children, we have calculated the transportation costs for the household as a whole. **Consumables** include the cost of food, water and other consumables around the health facility during the patient’s stay. Additionally, **dietary requirements** include the cost of dietary modifications needed by the patient to fully recover from the cholera episode, e.g. electrolytes, barley water, buttermilk, broths and other “special” food or drinks. These costs were reported directly by the patients (and caregivers) and were obtained from the household questionnaire.

**Indirect costs** encompass the productivity losses, which refer to those costs associated with the economic losses resulting from cholera morbidity, in other words, the days of missed work or absenteeism from work for the patient and/or their caregivers. Individual incomes were elicited directly from the study participants. However, where incomes were missing, they were predicted using as the average income of the sample and then adjusted for age in line with other cost of cholera illness papers [15, 22]. For adults, this was the average daily wage of the patients. For teenagers, it was half of the average adult patient’s daily wage and for the children, a quarter of the average daily wage of an adult patient.

### Health Facility Costs

The health facility costs include the various expenses incurred by the CTCs for treating a cholera patient. This cost category includes the **personnel** at the health facility, the **medical supplies** used to treat patients, the **medicine** used at the facility and facility **operational costs**.

**Health personnel** (staff) include the salaries of the personnel employed at the facilities for patient care. **Medical supplies** were the costs associated with the procurement of medical equipment and other necessary supplies to treat cholera. **Medicine** were the costs related to the medications used to treat patients with cholera. Lastly, **operational costs** (utilities) were those essential recurring costs for the facility to remain functional and provide its services (electricity and water bills). The cost of treatment of cholera to the health facilities was obtained by measuring these cost categories in the five selected cholera treatment centres.

### Data Analysis

We collected data using the ODK forms which were then inputted into Microsoft Excel. Data collection managers reviewed and cleaned entries. After data cleaning, we used Stata (version 14) to undertake the analyses. All monetary values were estimated in United States dollars (US$) at the 2023 rate.

Direct household costs were calculated as the sum of all costs incurred by the patient’s household during the cholera episode. Indirect costs were calculated as the cost of lost wages for both the patient and caregiver based on their self-reported missed workdays caused by the cholera episode. Wages were estimated from the self-reported salary income for patient and caregiver. This salary was pro-rated as a daily wage. Where salaries were not reported, we estimated them using a nearest neighbour imputation approach [23]. The reported monthly family income in this study was consistent with the findings a vulnerability assessment [24] in 2020 which reported the household monthly income of the families in Somalia. The nearest neighbours approach also reflected gender roles in labour market participation as well as earned income for those employed [25, 26].

The overall cholera cost to a household was calculated as the sum of all direct and indirect costs incurred during the cholera episode. The average household cost of a cholera episode was calculated as the sum of overall cholera cost of all households divided by the number of patients treated. We stratified the average household cost of a cholera episode by age group and sex.

The treatment centre cost of treating cholera was calculated by summing the expenditure on staff salaries, drugs, consumables and utilities (water and electricity). The cost for each health facility staff member (including doctors, nurses, health technicians, CHWs, and security and cleaning staff, if applicable) was calculated by multiplying the length of the stay of the patient at the health facility by the appropriate gross wage per hour, which was obtained from the interviews. The overall personnel cost was then calculated by summing these estimations. The cost of drugs and consumables was estimated through the interviews at the cholera treatment centre and with cholera experts in Somalia by first identifying the standard treatment based on the duration of the patient stay at the health facility and then multiplying the units of drugs and consumables used to treat a patient by its price. We obtained prices of drugs and consumables from the UNICEF supply catalogue [27]. Lastly, utility expenses (electricity and water bills) or operational costs were obtained from the five selected cholera treatment centres– the bills for 6 months were averaged to obtain a monthly average. One of the centres (Belethawo) operated in a rented building; therefore, the monthly rent was included for this health facility.

### Ethical Aspects

The research protocol was approved by the Federal Ministry of Health, Somalia. All the data were handled confidentially and depersonalized before analysis. Only the data collectors had access to this information and the researchers during the analysis. Data were stored in a password-protected server with access only for the researchers. We did not provide participants with any incentives to take part in this study and individual consent was obtained from all participants. In the case of participants younger than 18 years, consent was taken from their parents/caregivers. Data were anonymous at the beginning of the analysis.

## Results

### Patient Characteristics

Table 1 summarizes selected characteristics of the 400 patients in our study. Just over half (54.3%) of the sample were female and 71.8% were younger than 16 years, with 45.3% being 5 years or younger. Three quarters of the sample had not completed primary school. Most of the patients (82.0%) lived in urban settings and 12.5% lived in camps for internally displaced persons. Most patients (73.0%) had access to private drinking water sources. The great majority of patients (94.0%) had not received a cholera vaccine in the past. Our study included a large portion of children under 5 years old, reflecting the demographic distribution of cholera cases in Somalia. Consequently, we decided not to report schooling level since this age group is not typically engaged in formal education yet.

**Table 1:** Sociodemographic characteristics of the patients, Somalia

Characteristic	No.	% (*n* = 400)
**Sex**		
Female	217	54.3
Male	183	45.8
**Age group**, **in years**		
0–5	181	45.3
6–15	106	26.5
16–24	39	9.8
25–40	49	12.3
> 40	25	6.3
**District**		
Baidoa	40	10.0
Banadir	240	60.0
Belethawo	40	10.0
Jowhar	40	10.0
Kismayo	40	10.0
**Setting**		
Urban	328	82.0
Rural	22	5.5
IDP camp	50	12.5
**Drinking water source**		
Private	292	73.0
Public	108	27.0
**Household size**		
1–3	125	31.3
4–6	141	35.3
7–9	91	22.8
≥ 10	43	10.8
**Received a cholera vaccine**		
Yes	24	6.0
No	376	94.0

IDP: internally displaced person

The average household size was 5.5 persons (range 1–14). The average length of stay for cholera patients in our sample was 3.08 days, with patients missing almost 4 days of work or school on average.

### Household Costs

The total average cost of a cholera episode for a patient and their family was US$ 33.94. This amount includes US$17.12 in direct costs, such as transportation, medicine, dietary requirements among other necessities. The remaining US$ 16.82 represent indirect costs, which represent the lost wages for the patient and their caregivers due to missed workdays (Table 2). Of the direct costs, US$ 2.41 were medical and US$ 14.72 were non-medical. Direct medical costs are mostly covered by federal government of Somalia and development partners, explaining why the direct medical cost is low for the households, this cost component includes any additional expenses different than those covered by the health facilities; medicines (US $1.80) were the largest portion within this subcategory corresponding to medicines purchased by the households before and/or after their treatment at the health facilities. Transportation (US$ 6.02) was the highest direct non-medical cost, followed by consumables (US$ 4.91) and dietary requirements (US$ 3.79). Of the indirect costs, the average patient productivity loss was US$ 10.62, while the average caregiver productivity loss was US$ 6.20.

**Table 2:** Cost of a cholera episode for patients and their households, Somalia

Cost category	Average cost (SD)	Proportion of total household cost
**A. Direct medical costs**		
Consultation	0.04 (0.35)	0.00
Diagnostics	0.26 (1.27)	0.01
Hospitalization	0.32 (2.31)	0.01
Medicine	1.80 (7.04)	0.05
**Subtotal**	**2.41** (8.13)	0.07
**B. Direct non-medical costs**		
Consumables	4.91 (4.10)	0.14
Dietary requirements	3.79 (10.72)	0.11
Transportation	6.02 (10.11)	0.18
**Subtotal**	**14.72** (17.32)	0.43
**C. Total direct cost (A + B)**	**17.12** (22.24)	0.50
**D. Indirect costs**		
Patients’ productivity loss	10.62 (20.23)	0.31
Caregivers’ productivity loss	6.20 (10.58)	0.18
**Total indirect costs**	**16.82** (24.61)	0.50
**E. Total household cost of a cholera episode (C + D)**	**33.94**	1.00

SD: standard deviation

Based on our sample, we observed that caregivers were primarily part of the same household or other family members who provided care without formal compensation. While these caregivers did not receive a payment for their caregiving duties, the economic impact of their time and effort was accounted. We estimated the value of their lost time by calculating their lost productivity, which was assessed based on their self-reported missed days of work and their usual wage rates or the opportunity cost of their time.

Table 3 presents the average cost to a household of an episode of cholera by patient’s age group and sex. Patients aged 41 years and older incurred the highest mean total cost (US$ 73.90) while patients younger than 5 years had the lowest cost (US$ 21.02). Direct costs were highest for patients older than 40 years while indirect costs were highest for those aged 16–24 years. The wide variation across age groups costs could be explained by the younger groups in average typically requiring less intensive medical treatments. Indirect costs increased as age group was older, given that children contribute less to the household income, adults age groups had higher productivity losses. The mean total cost of a cholera episode was similar for females and males, US$ 33.87 and US$ 34.08, respectively. In average, male patients had slightly higher direct costs than female patients. The indirect cost for females was made up of the patient’s income reduction (US$ 10.07) and their caregiver’s productivity losses (US$ 7.92) (data not shown in table). For males, the patient’s income reduction was US$ 11.23 and their caregiver’s productivity losses were US$ 4.26. As a whole (patient and caregiver), indirect costs were higher for female patients, but we observed that the indirect cost for patients only (excluding caregiver costs) was higher for male patients, which could be explained by the higher proportion of men in the labor force of Somalia.

**Table 3:** Average cost of a cholera episode to households, by patient age group and sex, Somalia

Variable	Number of patients	Mean cost, in US$		
		Direct cost	Indirect cost	Total cost
**Age group, in years**				
0–5	181	12.8	8.22	21.02
6–15	106	16.12	14.97	31.09
16–24	39	18.64	42.52	61.16
25–40	49	22.96	29.29	52.16
> 40	25	38.84	35.06	73.90
**Sex**				
Female	217	15.88	17.99	33.87
Male	183	18.59	15.49	34.08

Cholera can significantly affect the livelihood of patients and their families, and the costs incurred during a cholera episode can force many families to adjust their finances and spending. On some occasions, families have to take extreme measures to compensate for the loss of income and the expenses imposed by the disease. Table 4 shows the adverse consequences faced by the families during the patient’s cholera episode and the measures they took to deal with the cholera episode. Most households (61.8%) used family savings to cover the costs of the cholera episode. Some families had to resort to borrowing money (17.5%), reducing household expenses (11.0%) and selling livestock at discount price (5.0%) while others had to take more drastic measures and sell their land and means of production.

**Table 4:** Household strategies to cope with the cholera episode, Somalia

Strategy used	No.	% (*n* = 400)
Borrowed money	70	17.5
Reduced household expenses	44	11.0
Sold agricultural products (tools and crops)	13	3.3
Sold jewellery and other assets	25	6.3
Sold land	5	1.3
Sold livestock or harvest at discount price	20	5.0
Used family savings	247	61.8
None	8	2.0

Note: Strategies were not mutually exclusive and some households used more than one strategy

### Treatment Centre Costs

The treatment centre costs analysis solely focused on direct costs (personnel, drugs and consumables, and utilities). Table 5 presents a breakdown of the average treatment cost of cholera for the three categories of cost stratified by the length of a patient’s stay/hospitalization. All costs increased with the length of the hospitalization. The average total cost of cholera for the health facility was US$ 82.65 per cholera episode. The cost for treating an outpatient was US$ 17.66. The cost for an inpatient hospitalized for 1–3 days was US$ 90.43 and for an inpatient hospitalized for > 3 days the cost was US$ 139.86.

**Table 5:** Average treatment cost per patient of cholera for the centres, by length of stay, Somalia

Cost category	Outpatient stay		Inpatient stay				Overall average	
	≈ 12 h		1–3 days		> 3 days			
	US$	% of cost	US$	% of cost	US$	% of cost	US$	% of cost
Personnel	14.54	82.3	72.69	80.4	116.30	83.2	67.84	82.1
Drugs, consumables	2.44	13.8	14.32	15.8	18.09	12.9	11.62	14.1
Utilities	0.68	3.9	3.42	3.8	5.47	3.9	3.19	3.9
**Total**	**17.66**	**100.0**	**90.43**	**100.0**	**139.86**	**100.0**	**82.65**	**100.0**

Personnel costs represented 82.1% of the average total cost of treatment, while drugs and consumables accounted for 14.1% and utilities for 3.9%.

Table 6 shows the average treatment costs on each of the five cholera treatment centres (Baidoa, Banadir, Belethawo, Jowhar and Kismayo).

**Table 6:** Average treatment cost of cholera by treatment facility

Treatment centre	Cost category	Outpatient stay	Hospital stay		Overall average
		≈ 12 h	1–3 days	> 3 days	
		US$	US$	US$	US$
Baidoa	Personnel	15.23	76.14	121.83	71.07
	Drugs, consumables	2.39	11.11	14.83	9.44
	Utilities	1.02	5.09	8.15	4.76
Banadir	Personnel	18.05	90.25	144.39	84.23
	Drugs, consumables	2.78	17.62	21.74	14.05
	Utilities	0.36	1.79	2.87	1.68
Belethawo	Personnel	18.91	94.55	151.29	88.25
	Drugs, consumables	2.78	17.62	21.74	14.05
	Utilities	0.60	2.99	4.78	2.79
Jowhar	Personnel	7.30	36.50	58.40	34.06
	Drugs, consumables	2.39	11.11	14.83	9.44
	Utilities	0.35	1.76	2.82	1.64
Kismayo	Personnel	13.20	66.00	105.61	61.60
	Drugs, consumables	1.88	14.14	17.30	11.11
	Utilities	1.09	5.47	8.75	5.10

The costs varied across health facilities. In terms of personnel costs, Belethawo (US$ 88.25) and Banadir (US$ 84.23) reported the highest costs. For the drugs and consumables costs per patient, Banadir and Belethawo recorded the highest expenses (each US$ 14.05) followed by Kismayo (US$ 11.11). Lastly, for utilities, Kismayo registered the highest cost at US$ 5.10, while Jowhar reported the lowest expenses (US$ 1.64).

As regards the average total cost per patient, Belethawo recorded the highest cost (US$ 105.09), followed by Banadir (US$ 99.96), Baidoa (US$ 85.25) and Kismayo (US$ 77.81). Jowhar recorded the lowest cost (US$ 45.14).

Despite CTCs providing a similar treatment for cholera cases, the CTCs reported slight differences in the drugs and consumables utilized when treating a patient. The personnel structure and their salaries also varied across CTCs. Furthermore, utilities expenses were understandably different from CTC to CTC based on their sizes and capacities. The differences in costs could be attributed to several factors. It also is important to note that the costs were calculated on a per patient basis. Since facility running costs as well as human resources costs were considered fixed, the number of patients treated at any facility would significantly affect the estimated cost per patient.

The overall average costs to households and health facilities was US$ 116.59 (US$ 33.94 for households and US$ 82.65 for health facilities). This means that averting one cholera episode can translate into substantial savings for households and health facilities. These findings carry direct policy implications and emphasize the need for prioritized investments in cholera prevention and control in Somalia.

## Discussion

Cost-of-illness studies are important tools to understand the economic burden of a disease or diseases and estimate the maximum amount that could potentially be saved or gained if a disease were to be eliminated. These studies have been pivotal in leading public health policy debates especially so in resource-constrained settings where the economic burden of a disease which is endemic and often results in explosive outbreaks owing to the poor social determinants remain unknown. While in the past a model-based study [14] provided valuable insights into the economic impact of cholera on Africa as a whole with estimates for several countries (including Somalia), our study offers a localized perspective specific to the country context in Somalia with a granular assessment of the economic cost of cholera for households and health facilities and considering the current context of the country and its healthcare dynamics.

As such, we have conducted this study to address a critical knowledge gap in a humanitarian crisis setting in Somalia. We believe that data coming out of such studies from a high-burden cholera countries like Somalia, a country also chronically affected by climatic shocks and other armed conflict and humanitarian crisis, can inform global policy discussions appropriately on achieving cholera elimination goals by the year 2030 in countries affected by humanitarian crisis. Our study also aims at advocating and presenting a case for improved and sustained investment on prevention and control strategies than to respond to endemic and epidemic cholera in high-burden countries. Our study, first of this kind in Somalia and possibly for other countries in humanitarian crisis, showcases that substantial cost-savings can be achieved by the government and international aid agencies if appropriate investments are made on cholera elimination. Our study also shows that high societal and health systems cost can be averted if cholera elimination goals are achieved in Somalia and other countries with week health system.

In our study, we estimated the cost associated with the treatment of cholera for health facilities and the patients (households). In our sample, 45% of the patients were children younger than 5 years, which aligns with the demographic distribution of cholera cases in the country. This high number of young children affected by cholera is concerning since research shows that early infancy exposure to cholera could have a greater long-term impact on adult height [28], suggesting that besides the immediate health risk, cholera can also have lasting effects on children growth and development. We have seen in our study that the economic burden on households extends beyond direct medical expenses to significant indirect costs, such as lost wages and disrupted education, as a single cholera case could lead to almost four (4) days of lost wages for the patient. This can result into serious economic consequences for the households, especially in a context where many families are engaged in informal employment and rely on daily wages for sustenance. This strain is even more acute in households where the patient or the caregiver is the primary breadwinner or contributes significantly to the family income. The negative impact also extends to missed education, as children affected by cholera may be unable to attend school due to illness or fear of contagion. Even short absences from school can have detrimental effects on educational achievement and incur huge societal cost [29, 30], especially in a low-income country like Somalia where educational resources are limited and the primary school enrolment rate is below 30% [31].

Our study found that the average cost to households affected by cholera was US$ 33.94 and the average health facility costs amounted to US$ 82.65 totalizing US$ 116.59. Our cost estimates are broadly comparable to those reported in previous cost of cholera illness studies, including Matlab (US$61.50), Kolkata (US$65.40) and Beira (US$ 77.20) in 2005 [22], Zanzibar (US$ 104) in 2009 [17],, and Malawi (US$125.30) in 2016 [20]. Furthermore, our estimates broadly align with the model-based estimation for Somalia, conducted by Mogasale et al. in 2021, of US$ 107.75 [14] and the average household cost of cholera in Bangladesh through a study conducted in 2013 (US$30.40) [15].

The expenses associated with medical treatment and care for cholera patients can exacerbate financial burdens, even in Somalia where cholera treatment is provided at a minimal cost through cholera treatment centres, as there might be other expenses related to transportation, special diets, and additional caregiving needs. More than half of the cases in our sample had to borrow money or use family savings to pay the costs associate with their episode of cholera, which consistent with the proportion reported by similar studies [20, 21]. Additionally, the stigma surrounding cholera can lead to social ostracization and discrimination against patients and their families, exacerbating the psychological toll of the disease [32]. The trauma endured by the family and in some cases the loss of a household member could compound these challenges, leaving lasting scars in the community. In our study we found that the households’ costs were higher for adult patients compared to children patients, primarily because of indirect costs, as adults experienced greater income and productivity losses than child patients. These results align with the findings of similar cost of illness studies conducted elsewhere [15, 20].

The negative impact of a cholera case extends beyond the household level as cholera has an economic impact on the local economy extending past the immediate healthcare costs and household expenses. Despite the illness lasting only a few days, short term diseases like cholera can be disruptive as productivity losses can ripple through various sectors, affecting businesses, production and economic output causing significant economic burden on countries where the disease is endemic [5, 33]. The total economic burden of cholera for Somalia was estimated to be US$3.29 M in 2015 (US$4.35 M in 2024) [14].

Cholera outbreaks could further strain the already challenged healthcare systems in Somalia, diverting resources away from other essential health services and leading to increased healthcare costs for both individuals and facilities. Health facilities costs varied across the five cholera treatment centres included in our study. These variations can be explained by their different personnel structures and salaries, the difference in the drugs and consumables and their utilities expenses, these differences are expected as it is the case in other studies analysing costs in more than one health facility [17].

Given the limited resources in Somalia, prioritizing cholera-prone areas could be a convenient way to optimize their use resources and efforts against cholera. As suggested in an exercise mapping the burden of cholera in sub-Saharan Africa [5], even though cholera occurs all over a country, prioritizing high-risk areas can substantially increase the efficiency of cholera control programmes [34].

The role of community health workers (CHWs) in cholera treatment and prevention in Somalia is crucial, especially given the challenges in accurately determining the true burden of cholera in communities. Many individuals who experience cholera symptoms do not visit health facilities [35], one of the reasons for not seeking professional healthcare being the perception that their episodes are not severe enough [36]. The CHWs can address this gap by actively engaging with the communities at the household level, promoting safe practices to avoid infection and improving community help-seeking behaviour by referring suspected cases to cholera treatment centres. Other experiences in the mobilization and training of CHWs have been implemented successfully and have proven to be efficient in cholera prevention and lifesaving treatment in resource-constrained setting with limited healthcare access [37]. Additionally, in the past, CHWs have been a reliable and common source of information during social mobilization activities for OCV campaigns in Somalia [38]. Among our sample, vaccination rates were notably low with only six (6) % of the patients having received a cholera vaccine in the past. The lack of immunity in a large portion of the population increases the susceptibility to infection, particularly in complex settings. Efficacy and effectiveness studies consistently demonstrate that OCVs prevent cholera by providing substantial direct protection and inducing herd protection among unvaccinated individuals [39, 40]. Furthermore, OCV campaigns are recognized as cost-effective interventions [17, 21, 41] in real world settings, including complex settings where poor sanitation, access to clean water and the presence of vulnerable populations are risk factors [42], similar to the current situation in Somalia. This implies that improving vaccination coverage could alleviate the economic and healthcare burdens on the communities affected by cholera.

Combining the services of cholera treatment centres with proactive actions, such as cholera monitoring, risk communication and community engagement activities through CHWs, WASH activities and OCV campaigns, could allow public health authorities and humanitarian organizations in Somalia to tailor vaccination strategies to the specific needs of the population and circumstances of a particular outbreak.

By providing estimates of the cost of illness related to cholera from the perspectives of health facilities and households, our results could be a valuable resource for public health policymakers planning interventions in countries with similar settings. This study contributes to the existing literature in the field by bridging informational gaps and helping to understand the economic ramifications of cholera. Lastly, by quantifying a true cost of cholera our study can act as an investment case for cholera interventions, emphasizing the economic rationale for cholera programmes.

### Limitations

The retrospective interview approach used for data collection could result in recall bias since the interviews were conducted after the patients completed their treatment to allow for all costs during the cholera episode to be considered. To mitigate this risk and still allow the patients to complete their treatments, interviews were conducted 15–20 days after the patients were out of the cholera treatment centre. Additionally, we carefully designed the questionnaire, limited the time frame to the cholera episode, avoided leading questions and validated the information through external sources.

While we purposefully selected the cholera treatment centres based on caseloads and accessibility and security, we ensured the representativeness of our data by employing Kish Leslie formula to determine our sample size, using proportionate sampling across the selected districts and by randomly selecting the participants in our sample.

In our sample, only two out of the 400 patients died as a result of cholera. Therefore, our analysis did not consider the household costs associated with fatal cholera cases. Roughly two fifths of our sample were children. Based on our analysis, the cost of a cholera episode in a child is lower than in an adult. Thus, care must be taken when extrapolating the cost estimates in this analysis to the national level. As most cholera cases in Somalia occur in children younger than 5 years, estimates presented in this analysis likely overestimate the national average cost. Indirect costs were calculated as the number of days missed from work. No intangible costs, such as school days missed, stigma or reduction in productivity, were considered.

The costs incurred in the establishment of the cholera treatment centres, such as building and land used, were excluded from the analysis since they vary by size and type of structure. Including these expenses would result in a higher cost per cholera patient for the health facilities. The costs associated with the procurement and delivery of the resources used to treat the patients were also not included in the analysis, since many treatment centres are funded by donors and the study focused on the cost per unit of those resources and not the administrative process. Only direct treatment costs and recurring costs were considered in the analysis. Had other costs been considered in the analysis, the health facility cost of treating a cholera patient would likely have been higher than our estimate.

## Conclusion

This is the first study from a country with fragile and conflict-affected situations assessing the cost of illness of cholera at both household and health facility level.

Our study aimed to assess the economic consequences of cholera in Somalia, on both families and health facilities. Our findings suggest that preventing one cholera episode in Somalia could avert substantial losses of US$ 116.59 per cholera episode– US$ 33.94 at the household level and US$ 82.65 at the cholera treatment centre level.

Our findings highlight that the costs of cholera go beyond those incurred by the healthcare payer and that significant costs are incurred by patients both directly and indirectly. If these losses are considered, preventing cholera cases could reduce economic costs to the provider, the individual and the broader Somali economy. Our study sheds light on some often overlooked, yet significant, hidden costs associated with cholera, which encompass a wide range of intangible consequences. Thus, our results strengthen the case for greater investment in cholera case prevention.

## Electronic Supplementary Material

Below is the link to the electronic supplementary material.

Supplementary Material 1

Supplementary Material 2

## Data Availability

Data is provided within the manuscript and as supplementary information and additional files.
