# Supplementary material for: Cost of Cholera for Households and Health Facilities, Somalia
Source: J Epidemiol Glob Health. 2024 Jul 18;14(3):1219–30. doi: 10.1007/s44197-024-00278-6 (PMC11442814; doi:10.1007/s44197-024-00278-6)
Supplement: Supplementary file 1 — Supplementary Material 1 [file 44197_2024_278_MOESM1_ESM.docx]

**Cost of Illness (COI) of treating Cholera at a Health Facility in Somalia.**

***Questionnaire for Collecting Costs of Treating Cholera at a Health Facility***

## Module 0: Introduction and Consent

Good morning, my name is ____________________________________ [*interviewer*]. We are working with WHO Somalia to collect information about the costs of illness related to cholera. The goal of this interview is to acquire the expenditures incurred by a facility due to cholera. Before you decide to participate in this study, I must read this.

**Participation in this survey is entirely voluntary, and all information you submit will be kept in secret. Your identities will not be published. The information will only be used for estimating the cost of illness related to cholera and cost-effectiveness research. This investigation intends to calculate the expenses paid by you and your family during the cholera outbreak.**

**During this interview, feel free to clarify any points that are unclear. There will be no penalty for refusing to participate or withdrawing from the study at any time during the interview. However, we encourage the facilities to complete the study since the information you offer is very valuable, it will allow us to estimate the costs of preventing and dealing with future cholera outbreaks.**

If you have any questions or concerns about this assessment, please feel free to contact _________________________ [*designated costing contact*] and telephone number___________________________________.

**Consent to Participate:**

I have read [heard] the information provided above and i understand it. I have been allowed to ask questions and all of my questions have been answered to my satisfaction.

**_____________________________ ____________________________**

**Name of Respondent Respondent’s contact**

**_____________________________ ___________________________**

**Name of Interviewer Interviewer’s contact**

## Module 1: General Facility Information

This questionnaire is to be completed by interviewing health care providers in health facilities with Cholera Treatment Centres (CTCs). Interviewees must have been in contact and involved with providing treatment and care for cholera patients during the most recent cholera outbreak.

### Section A: Identification of facility

A1. Date of the interview: ___________________________________________________

A2. Health facility name: ___________________________________________________

A3. District:­­­­­­­­­­­­­­­­­­­­___________________________

### Section B: Identification of respondent

B1. First and last name of respondent: _________________________________________

B2. Title of respondent: _____________________________________________________

B3. Contact number of respondent: ____________________________________________

## Module 2: Costs of Treating Cholera

### Section C: Cholera Cases

| **In this section, we want to know the number of cholera cases that were treated, and the length of hospital stay** |
| --- |
| C1. How many cases were treated in this health facility in the last 6 months (MAY-OCTOBER)  Outpatient (Same Day): _________________ cases  Stayed 1-3 days at the health facility: ____________________ cases  Stayed more than 3 days at the health facility: ______________ cases.  TOTAL________  Deaths: __________________ cases |

| **In this section, we want to know the number of cholera cases that were treated, and the length of hospital stay** |
| --- |
| C2. How many cases were treated in this health facility in OCTOBER?  Outpatient (Same Day): _________________ cases  Stayed 1-3 days at the health facility: ____________________ cases  Stayed more than 3 days at the health facility: ______________ cases.  TOTAL________  Deaths: __________________ cases |

This section will collect information about costs incurred by your health facility to treat patients who suffered from cholera. (Please review patient clinical files for detailed information).

### Section D: Resources used for OUTPATIENT TREATMENT (LESS than one day)

D1. Please, specify for **drugs and consumable materials** used, quantity and unit cost. Use the empty cells to inform the medicines we have not listed and have been used.

| **Drug Name** | **Unit presentation of drug** | **Quantity used** | **Unit cost** |
| --- | --- | --- | --- |
| Oral rehydration salts | Bag |  |  |
| Doxycycline |  |  |  |
| Azithromycin |  |  |  |
| Zinc supplementation |  |  |  |
| Erythromycin |  |  |  |
| Examination Gloves |  |  |  |
| Personal Protective Equipment (PPEs) |  |  |  |
| Disinfectants |  |  |  |
| Syringe and needle |  |  |  |
| Stool Sample Test |  |  |  |
| Carry-plair |  |  |  |
| IV giving set |  |  |  |
| Cannula |  |  |  |
| **Other (please specify) ______________** |  |  |  |
| **Other (please specify) ______________** |  |  |  |

### Section E: Resources used for INPATIENT TREATMENT

E1. Please, specify for **drugs and consumable materials** used, quantity and unit cost. Use the empty cells to inform the medicines we have not listed and have been used.

| **Drug Name** | **Unit presentation of drug** | **Quantity used** | **Unit cost** |
| --- | --- | --- | --- |
| Oral rehydration salts | Bag |  |  |
| Ringers Lactate |  |  |  |
| Doxycline |  |  |  |
| Azithromycin |  |  |  |
| Zinc supplementation |  |  |  |
| Erythromycin |  |  |  |
| Personal Protective Equipment (PPEs) |  |  |  |
| Disinfectants |  |  |  |
| Examination Gloves |  |  |  |
| Syringe and needle |  |  |  |
| Stool Sample Test |  |  |  |
| Cholera RDT |  |  |  |
| Carry-plair |  |  |  |
| IV giving set |  |  |  |
| Cannula |  |  |  |
| **Other (please specify) ______________** |  |  |  |
| **Other (please specify) ______________** |  |  |  |

### Section F: Personnel working in the CTC

F1. on average, which of the following **health facility professionals** work and treat cholera in the CTC.

| Health professionals | How many participates to **inpatient** cholera treatment? | Salary **(PLEASE SPECIFY IF SALARY MONTH OR PER DAY)** | How many work in the CTC ? |
| --- | --- | --- | --- |
| Doctor | Yes  No  N |  |  |
| Nurse | Yes  No  N |  |  |
| Health surveillance assitant | Yes  No  N |  |  |
| Laboratory technologist | Yes  No  N |  |  |
| Community Health Workers | Yes  No  N |  |  |
| Cleaner | Yes  No  N |  |  |
| Security person | Yes  No  N |  |  |
| Other*, specify:  ________________________ | Yes  No  N |  |  |

### Section G: Expenses in the Cholera Treatment Center (CTC)

G1. on average, what are tipically the expenses for the CTC being able to operate.

Electricity USD/per month. Provide receipts for the last six months, if available.

Water USD/per month. Provide receipts for the last six months, if available.
