# Supplementary material for: Cost of Cholera for Households and Health Facilities, Somalia
Source: J Epidemiol Glob Health. 2024 Jul 18;14(3):1219–30. doi: 10.1007/s44197-024-00278-6 (PMC11442814; doi:10.1007/s44197-024-00278-6)
Supplement: Supplementary file 2 — Supplementary Material 2 [file 44197_2024_278_MOESM2_ESM.docx]

**Cost of illness (COI) related to Cholera in Somalia**

***Questionnaire for Collecting Costs of Cholera Illness at a Household***

## Module 1: Introduction and Consent

Good morning, my name is ____________________________________ [*interviewer*]. We are working with WHO Somalia to collect information about the costs of illness related to cholera. The purpose of this interview is to obtain costs borne by your household during a cholera episode by a member of your household. Before you decide to take part in this study, we would like to explain the purpose of the study.

The goal of this interview is to acquire the expenditures incurred by you and your family caused by cholera to you or a member of your family. Before you decide to participate in this study, I must read this.

**Participation in this survey is entirely voluntary, and all information you submit will be kept in secret. Your identities will not be published. The information will only be used for estimating the cost of illness related to cholera and cost research. This investigation intends to calculate the expenses paid by you and your family during the cholera episode.**

**During this interview, feel free to clarify any points that are unclear. There will be no penalty for refusing to participate or withdrawing from the study at any time during the interview. However, we encourage the participants to complete the study since the information you offer is very valuable, it will allow us to estimate the costs of preventing and dealing with future cholera outbreaks.**

If you have any questions or concerns about this assessment, please feel free to contact ________________________________ [*provide name of designated costing contact*] and telephone number ­­­­­­­­­­­­­­­­­­­­___________________________________.

**Consent to Participate:**

I have understood the information provided above. I have been allowed to ask questions and all my questions have been answered to my satisfaction.

**_____________________________ ____________________________**

**Name of Respondent Respondent’s Contact**

**_____________________________ _____________________________**

**Name of Interviewer Interviewer’s contact**

# Module 2: General Information

## Section A: Identification of the participant

A1. Identification number in the study

(*Interviewers make sure to concisely report the ID number you gave on the consent form*)

A2. Date of the interview: (DD/MM/YY) ______________________________

A3. Where is the patient from (where does the patient live)?

Kismayo (1) Banadir (2) Banadir (3)

Belethawo (4) Jowhar(5) Other, specify (6)

A4. Is the participant living in a rural or an urban area?

Rural area (1) Urban area (2) IDP(3)

## Section B: Patient’s demographic characteristics

B1. What is the sex of the patient? Male (1) Female (2)

B2. How old is the patient?

B3. What is the occupation of the patient? ______________________

B4. Could you please tell me about your relationship with the patient?

Self (patient) (1) Parent (2) Grandparent (3)

Sibling (4) Partner (5) Other (6) ………………

B5. What is the highest level of schooling (the patient) has attained?

Primary (1) Secondary (2)

Tertiary (College/ University) (3) No formal schooling (4)

B6. What is (the patient) marital status?

Single (1) Divorced (2)

Married (3) Separated (4)

Widowed (5) Other, specify (6) ……………………………

B7. What is the main source of drinking water in your household?

Private water source (1) Public water source (2)

B8. What type of toilet do you have at home?

Flushing toilet (1) Open defecation (2)

Pit latrine (3) Other, specify (4) ……………………………

B9. How many persons are there in your household?

Kids (under 16) Adults (over 16) **Total**

## Section C: Patient’s treatment in health facility

**First, I would like to know how much you (the patient) had to spend on any healthcare services against cholera.**

C1. How many days were you (the patient) sick with diarrhea and vomiting before you visited the health center/ CTC?

Days

C2. Did you receive any treatment for the diarrhea and vomiting before visited the health center/CTC?

Yes (1)

No (2)

Do not know (3)

C3. Where else did you seek treatment for the diarrhea and vomiting before visited the health center/CTC? (Respondent can select more than one response)

District hospital (1) Health center/post (2) Cholera treatment center (3)

Cholera treatment unit (4) At home (5) Other, specify (6) …………………………………………

C4. When did the patient visited the health center/CTC? ______________________________(MM/YY)

C5. Have you (the patient) ever received a cholera vaccine?

Yes (1) when? ______________________________(MM/YY)

No (2)

*I would like to ask questions regarding the* ***visits*** *you have done in seeking care for cholera illness.*

| **Item** | **Quantity** | **Price (USD)** |
| --- | --- | --- |
| Food |  |  |
| Water |  |  |
| Special Diet |  |  |
| Item A |  |  |
| Item B |  |  |

C6. Did you (the patient) buy food and/or water for yourself (the patient) during your visit?

Yes (1) No (2)

Don’t remember (3) Don’t know (4)

C7. Did you (the patient) pay any consultation fee (to doctors, nurses) during your visit?

Yes (1) No (2)

Don’t remember (3) Don’t know (4)

C8. How much did you pay for consultations?

USD (*don’t know =XXXXXX)*

C9. Did you (or the patient) pay for any medicine/drugs during your visit?

Yes (1) No (2)

Don’t remember (3) Don’t know (4)

C10. How much did you pay for medicine/drugs?

USD (*don’t know=XXXXXX)*

C11. During the first visit, did you (or the patient) pay any diagnostic/lab fee?

Yes (1) No (2)

Don’t remember (3) Don’t know (4)

C12. How much did you pay for diagnostic/lab tests?

USD (*don’t know=XXXXXX)*

C13. During the first visit, did you (or the patient) pay any fee for transportation (from your

house to the first facility and back to your house)?

Yes (1) No (2)

Don’t remember (3) Don’t know (4)

C14. How much did you pay for the round trip to your house?

USD (*don’t know=XXXXXX)*

C15. During the first visit, did you (or the patient) pay any fee for hospitalization?

Yes (1) No (2)

Don’t remember (3) Don’t know (4)

C16. How much did you pay for hospitalization?

USD (*don’t know=XXXXXX)*

C17. Did you (or the patient) pay any other fee outside what we have mentioned above?

Yes (1) No (2)

Don’t remember (3) Don’t know (4)

C18. Please specify what you paid for?

…………………………………………………………………………………..

C19. How much did it cost? USD (*don’t know=XXXXXX)*

C20. In case you (or the patient) do not remember costs borne during the visit to the center, can you estimate how much you totally spent during your visit?

0-5 USD (1) 6-10 USD (2) 11-15 USD (3)

15-20 USD (4) over 20USD (5)

C21. What was the main means of transportation you (or the patient) used during your visit?

On foot/walking (1) Bicycle (2) Taxi (3)

Motorcycle (4) Minibus (5) Private car (6)

Other (7) Specify…………………………………

C22. How long did you (the patient) take to get to the place of your visit **(consider round trip)**?

Minutes (*don’t know=979)*

C23. Excluding (the patient), how many people in total accompanied you (the patient) to the place of the visit?

Number (0) if the patient was not accompanied)

C24. On average, how much did a round trip cost to each accompany person (*approximate please*)?

USD (*don’t know=XXXXXX; no expense=000000)*

C2. How long did you stay at the health facility/CTC?

**If less than one day**, how many hours

**If more than one day,** how many days_

**Section D: Indirect costs: Patient productivity losses & opportunity costs**

*Now, I would like to ask you about the ways you (or the patient’s) cholera illness affected those.*

*around you. Sometimes, people with cholera feel so sick that they cannot perform any of their usual*

*activities. Instead, they have to rest and stay in bed. Other people around the patient may not be as*

*sick, and can still do their usual activities. But they may not be able to perform their activities as*

*well as normal because they may have to help or take care of the patient*.

*When you answer these questions, please think about the entire time period when you were sick with*

*cholera, starting before you (or the patient) went to the health facility for diagnosis*.

D1. In total, how many days were you (or the patient) sick with cholera since the symptoms started? Days (*Don’t know=XX ; Not applicable=NA*)

D2. How many days were you (or the patient) **completely unable** to do any of your usual activities while sick? Days (*Don’t know=XX ; Not applicable=NA*)

D3. How many days were you (or the patient) absent from work/school?

Days (*Don’t know=XX; Not applicable=NA*)

D4. What would you (or the patient) be mainly doing if you had not been sick?

Working for an employer, informal (1) Working for self (2)

Government worker (3) Private worker, formal (4)

Fishing (5) Agriculture (6)

Housework (cooking, cleaning, etc) (7) Going to school (8)

Leisure or play time (9) Private worker, informal (10)

Studying (11) Unemployed (12)

Other, specify (13) ……………………

D5. Do you (or the patient) work for a wage (cash or in kind)?

Yes, paid in cash (1) Yes, paid in-kind (2) **D7**  No (3)

D6. How much are you paid per day? USD

(*Don’t know=XXXXX ; Refusal=ZZZZZ*)

D7. **IF PAID IN-KIND**, Please specify the approximate value per day? USD

(*Don’t know=XXXXX ; Refusal=ZZZZZZ*)

D8. If you work for a wage, did your (or the patient’s) employer or boss pay for the days you were sick but did not come to work?

Yes (1) No (2) Don’t know (9)

D9. How many sick days did your (or the patient’s) employer or boss pay for you?

Days (*Don’t know=XX ; Not applicable=NA*)

D10. In total, what is your (or the patient’s) monthly income USD

(*Don’t know=XXXXXX ; Refusal=ZZZZZZ*)

0-50 USD (1) 51-100 USD (2) 101-200 USD(3)

200- 300 USD (4) over 300 USD (5)

D11. In total, how many days of income did you (or the patient’s) lose because of this illness since it started?

Days (*Don’t know=XX; Patient did not return to work=YY; Not applicable=NA*

D12. What is the monthly income of your family?

USD (*Don’t know=XXXXXX ; Refusal=ZZZZZZ*)

D13. Who in the family generate income for the family? (Choose as many as they work) **Give the number of brothers and sisters that provide income.**

Self (patient) Mother Father Brothers (number: _____)

Sisters (number: _____) Grandparents (number: _____) Other, specify …………………

**Section E: Indirect costs: Caregivers & Substitute labor costs**

*Now I would like to understand if anyone helped you (or the patient) while you were sick. This includes 2 types of people:*

1. *Someone who may have been paid to complete your usual tasks for you*
2. *Someone who had to help take care of you (or the patient) while you were sick*

E1. Were you (or the patient) so sick with cholera that someone had to help you (or the patient)

in any way?

Yes (1) No (2) Don’t know (9)

E2. In total, how many people cared for you (or the patient) or did your usual activities for you while you were sick with cholera?

people (*Don’t know=XX*)

*Questions* ***E3 to E12*** *record productivity losses for the* ***helper/caretaker***

E3. What is (the patient) relationship with the person who helped (the patient) while sick?

Mother (1) Father (2) Husband/Partner (3) Wife/Partner (4)

Brother (5) Sister (6) **Hired help (7)** Grandparent (8)

Neighbor (9) Friend (10) Other, specify (11) ……………

E4. Is this helper a member of your household?

Yes (1) No (2) Don’t know (9)

E5. How old is this helper/caretaker?

years (*Don’t know=97* )

E6. How many days or hours did he/she helped you (or the patient)?

Days (*Don’t know=XX*) or Hours (*Don’t know=XXXX*)

E7. Was this helper paid to help you (or the patient)?

Yes (1) No (2) Don’t know (9)

E8. How much was this helper/caretaker **paid per day** to help the patient?

(*Don’t know=XXXXX ; refusal=ZZZZZZ*)

E9. How many days or hours did the helper cut back on his/her own activities?

Days (*Don’t know=97*) or Hours (*Don’t know=9797*)

E10. Was the helper able to do some or none of his/her own usual activities?

Able to do some of his/her usual activities (1)

None of of his/her usual activities (2)

Do not know (9)

E11. What would the helper have been doing mainly if they had not been caring for the sick patient?

Working for an employer, informal (1) Working for self (2)

Government worker (3) Private worker, formal (4)

Housework (cooking, cleaning…) (5) Going to school (6)

Studying (7) Other, specify (7) ……………………………

E12. If helper/caretaker works for a wage, how much is s/he normally paid per day for his/her?

USD (*Don’t know=XXXXXX ; refusal=ZZZZZZ*

E13. What is normally the monthly income of the helper/caretaker?

USD (*Don’t know=XXXXXX ; refusal=ZZZZZZ*

0-1000 USD (1) 1001-5000 USD(2) 5001-10,000 USD (3)

10,001-2,000 USD(4) over 20,000 USD (5)

**Section F: Financial impact of cholera and household socioeconomic status**

*There a few more questions I would like to ask you to understand how this cholera event impacted your (or the patient’s) financial situation*

F1. Did you (or the patient) or your household have to adopt some coping strategies in order to

pay for your (or the patient) healthcare because of cholera illness ?

Yes (1) No (2) Don’t know (9)

F2. Which strategy(ies) did you (or the patient) or the household adopted (*multiple choices allowed*)?

Sold land Yes (1) No (2)

Sold livestock or harvest Yes (1) No (2)

Sold agricultural products or tools Yes (1) No (2)

Sold household jewelry and assets (*bicycle, TV, clothes,)*  Yes (1) No (2)

Borrow money (*from household members and friends*) Yes (1) No (2)

Used savings Yes (1) No (2)

Reducing household expenses Yes (1) No (2)

F3. Compared to before you (the patient) visited the health facility, how is your (the patient) physical wellness today?

Worse (1) The same (2) Better (3)

**Section G: Costs associated with death from cholera**

Interviewer only proceed to this section, if the patient is dead.

Now, my coming question may evoke a bad memory to you as I will talk about the death of the member of your family who suffered from cholera

G1. What your (the patient) health status today?

Recovered from cholera (1). Still sick with cholera (2)

Sick with other disease (3) Deceased (4)

We are sorry getting to know that the patient passed away because of cholera. We

would like to ask a few more question on his burial. We understand these may evoke in you some negative feelings. Please accept our apologizes. Can we continue?

G2. How old was the patient at the time of death?

Age at death

G3. In total, how much did your family had to pay to cover funeral expenses (food, mourners, etc)?

USD (*don’t know=XXXXXX; no expense=000000; NA=NA)*

G4. In total, how much did your family had to spend at the morgue?

USD (*don’t know=NA; no expense=000000; NA=NA)*

G5. In total, how much did your family had to spend for the transportation of the dead person to the cemetery?

USD (*don’t know=XXXXXXX; no expense=000000; NA=NA)*

G6. In total, how much did your family had to spend for the coffin and the grave?

USD (*don’t know=XXXXXX; no expense=000000; NA=NA)*

Remarks………………………………………………………………………………………………

……………………………………………………………………………………………………………

Date of questionnaire review by the supervisor (if any) (DD/MM/YY) ……………………….

Supervisor signing…….………………………………………………………………….
